# Genome-wide landscape of DNA methylomes and their relationship with mRNA and miRNA transcriptomes in oxidative and glycolytic skeletal muscles

**DOI:** 10.1038/srep32186

**Published:** 2016-08-26

**Authors:** Linyuan Shen, Jingjing Du, Yudong Xia, Zhendong Tan, Yuhua Fu, Qiong Yang, Xuewei Li, Guoqing Tang, Yanzhi Jiang, Jinyong Wang, Mingzhou Li, Shunhua Zhang, Li Zhu

**Affiliations:** 1 College of Animal Science and Technology, Sichuan Agricultural University, Chengdu, Sichuan, China; 2 E-GENE, Shenzhen, Guangdong, China; 3 Key Laboratory of Agricultural Animal Genetics, Breeding, and Reproduction of Ministry of Education & Key Laboratory of Swine Genetics, Huazhong Agricultural University, Wuhan, China; 4 Department of Animal Husbandry and Veterinary Medicine, Chengdu Agricultural College, Chengdu, Sichuan, China; 5 Department of Biology, College of Life and Science, Sichuan Agricultural University, Chengdu, Sichuan, China; 6 Chongqing Academy of Animal Science, Rongchang, Chongqing, China

## Abstract

The physiological, biochemical and functional differences between oxidative and glycolytic muscles play important roles in human metabolic health and in animal meat quality. To explore these differences, we determined the genome-wide landscape of DNA methylomes and their relationship with the mRNA and miRNA transcriptomes of the oxidative muscle *psoas major* (PMM) and the glycolytic muscle *longissimus dorsi* (LDM). We observed the hypo-methylation of sub-telomeric regions. A high mitochondrial content contributed to fast replicative senescence in PMM. The differentially methylated regions (DMRs) in promoters (478) and gene bodies (5,718) were mainly enriched in GTPase regulator activity and signaling cascade-mediated pathways. Integration analysis revealed that the methylation status within gene promoters (or gene bodies) and miRNA promoters was negatively correlated with mRNA and miRNA expression, respectively. Numerous genes were closely related to distinct phenotypic traits between LDM and PMM. For example, the hyper-methylation and down-regulation of *HK-2* and *PFKFB4* were related to decrease glycolytic potential in PMM. In addition, promoter hypo-methylation and the up-regulation of miR-378 silenced the expression of the target genes and promoted capillary biosynthesis in PMM. Together, these results improve understanding of muscle metabolism and development from genomic and epigenetic perspectives.

Skeletal muscle is one of the body’s major organs and accounts for approximately 40% of body mass in mammals^1^. It has important roles in exercise and energy metabolism^2^. The functional diversity of skeletal muscle is closely associated with the highly heterogeneous composition of muscle fibers. In general, muscle fibers are divided into four different myosin heavy chain (MyHC) isoforms. Type I and Type IIa are oxidative (or red) fibers, whereas Type IIb and IIx fibers are glycolytic (or white) and intermediate fibers, respectively^3^,^4^. Oxidative fibers are highly enriched with capillaries, myoglobin, lipids, and mitochondria, and have a lower glycogen concentration and glycolytic enzyme activity than do glycolytic fibers. Thus, oxidative muscle can undergo chronic contractile activity with less fatigue than glycolytic muscle^5^,^6^ Moreover, the differences in muscle fiber types are closely related to metabolic health and disease. Daugaard *et al*. (2000) have found that insulin-stimulated glucose transport is positively correlated with the proportion of oxidative fibers in muscle tissue^7^. Goodman *et al*. (2012) have shown that glycolytic muscle fibers have a greater capacity for atrophy than do oxidative fibers^8^. In mammals, muscle fiber composition influences the conversion of muscle to meat and metabolism after death, thereby influencing meat quality. For example, the percentage of Type IIb fibers is negatively associated with pH_45_ (45 min postmortem), and Type I and IIa are negatively associated with drip loss^9^.

Oxidative and glycolytic muscles exhibit two extremes of the metabolic patterns of muscle tissue and serve as ideal models to study the genetic mechanism underlying the diverse phenotype of muscles. Using high throughput sequencing techniques, Campbell *et al*. (2001) have identified 49 differentially expressed mRNA sequences between white quadriceps muscle (glycolytic) and the red soleus muscle (oxidative) of mice^10^. Transcriptional analysis between the *longissimus dorsi* (glycolytic) and *soleus* (oxidative) skeletal muscles of pigs revealed 28 signaling pathways, including MAPK and Wnt pathways, which corresponded to the different phenotypic properties of muscle^11^. Liu *et al*. (2013), in a microRNA transcriptome analysis, have identified 173 miRNAs that are differentially expressed between glycolytic and oxidative muscles and are involved in muscle structural development and regeneration^12^. However, posttranscriptional modifications (changing the expression of mRNA and miRNA) alone do not adequately explain the mechanisms of the different phenotypic properties of muscle. In eukaryotes, DNA methylation is an important epigenetic modification that is dynamic and can alter gene expression in response to the environment and developmental status without changing DNA sequences^13^. Jin *et al*. (2014) have reported a genome-wide DNA methylation analysis comparing young and middle-aged muscles and have found that different methylation genes are involved in proteolysis and protein catabolic processes^14^. Nevertheless, little is known regarding the differences in the DNA methylomes of oxidative and glycolytic muscle. The present study reports an integrated genome-wide analysis of DNA methylation and mRNA and miRNA transcriptome profiling of pig *psoas major* (oxidative) and *longissimus dorsi* (glycolytic) muscles. This work should contribute to the understanding of both human muscle health and the improvement of the meat quality of livestock.

## Materials and methods

### Ethics Statement

Animal care and experiments were conducted according to the guidelines established by the Regulations for the Administration of Affairs Concerning Experimental Animals (Ministry of Science and Technology, China, revised in June 2004) and approved by the Institutional Animal Care and Use Committee in College of Animal Science and Technology, Sichuan Agricultural University, Sichuan, China under permit No. DKY- DKY-2014-18.

### Animals and tissue collection

Three female Landrace pigs were used. The pigs were fed twice daily from weaning (28 days old) to slaughter (210 days old) with a formula meeting the National Research Council (NRC 1998) recommendations for different growth phases and were given water *ad libitum*. Approximately 24 h before slaughter, the feed was removed, but the *ad libitum* access to water was maintained. The pigs were slaughtered by electrical stunning followed by exsanguination. The carcasses were then scalded and rinsed. Muscle samples were obtained from the intermediate section of the *longissimus dorsi* and *psoas major* immediately after exsanguination and rapidly frozen in liquid nitrogen for sequencing analysis. Samples were obtained from the front and terminal ends of the muscles for the measurement of meat quality traits.

### Measurement of LDM and PMM phenotypes

The myofiber area and myofiber type ratio were measured using ATPase staining as previously described^15^. After staining, fast fibers were a dark color, whereas Type II fibers were light. The myofiber type ratio was determined by the area of fast fibers divided by the area of slow fibers for 100 randomly chosen fields of vision. The mean area of fast and slow fibers was taken as the myofiber area of muscle. The fatty acid composition was quantified using a GC-14C gas chromatograph (Shimadzu) according to the method described by Qin *et al*^16^. All meat quality trait measurements were performed according to our previously described methods^6^.

### Methylated DNA immunoprecipitation sequencing

The *longissimus dorsi* and *psoas major* muscles from all three pigs were used to construct MeDIP DNA libraries. Briefly, approximately 5 μg of DNA extracted using a DNeasy Blood & Tissue Kit (Qiagen) was sonicated to 100–500-bp fragments with a Bioruptor sonicator (Diagenode). The fragments were ligated with adaptors and immunoprecipitated with a monoclonal anti-methylcytidine antibody. The enriched fragments with methylation and 10% input DNA were purified and amplified through adaptor-mediated PCR. Then, the MeDIP libraries were subjected to paired-end sequencing using an Illumina HiSeq 2000 and a 50-bp read length. Raw sequencing data were processed with the Illumina base-calling pipeline. Methylated DNA immunoprecipitation sequencing (MeDIP-seq) data have been deposited in the NCBI Gene Expression Omnibus under the GEO accession number GSE81759.

### Analysis of MeDIP-seq data and identification of DMRs

We filtered out low-quality reads that contained more than 5 ‘N’s and more than 50% of the sequences with a low quality value (Phred score < 5). The sequencing reads were aligned to the pig reference genome (Sscrofa 10.2), allowing up to four mismatches, by using SOAP2 (Version 2.21)^17^. The reads that mapped to multiple genomic locations were regarded as duplicates and were therefore selected only once. To avoid stochastic sampling drift, we filtered out CpG sites that were covered by less than a 10 read depth when performing subsequent analysis. Furthermore, we classified all the genomic regions into 24 genomic elements while annotating the reads into genomic locations, according to a previous study^14^. The DMR identification method was used as previously described^14^. The normality and equal variance of the read depth at each CpG in PMM and LDM were tested using Bartlett’s test (significantly different if *P* > 0.05). Subsequently, a parametric (passing Bartlett’s test) or non-parametric test (failing Bartlett’s test) was used to select highly variable CpGs (*P* < 0.01) as seed sites for candidate DMRs. Then, a site 3’ downstream adjacent to the CpG was incorporated into the seed CpGs (up to 200 bp) and the new seed CpGs repeatedly adjacent to the next CpG until a low-variance CpG (*P* > 0.01) appeared, which was allowed to be up to 2 kb from the seed CpG. If five or more CpGs in a genomic region had significantly different (*P* < 0.01) read depths between LDM and PMM, this result was considered to represent a DMR. The *P* values for the DMRs were also corrected using the Benjamini-Hochberg method (FDR < 0.01, 1,000 permutations).

### Measurement of mitochondrial DNA copy number, telomere length and gene expression

The relative mitochondria copy numbers were determined by q-PCR, which was designed to detect cytochrome oxidase 1 (*COX1*) and glucagon gene (*GCG*) for mitochondrial DNA (mtDNA) and nucleic DNA, respectively. The doubled ratio of COX1 to GCG within each sample was used to calculate mtDNA content. The relative telomere length had a positive correlation with the ratio of the telomere repeat (TTAGGG) copy number to the single copy gene copy number, as measured by two separate PCR reactions, as previously described, using a telomeric region primer (T) and a primer for a reference single copy nuclear gene (*GCG*, Single copy, S)^18^. The telomere (T) signal was normalized to the signal from the single-copy (S) gene to generate a T/S ratio indicative of the relative telomere length (all the primers are shown in Supplementary Table S1). All q-PCR reactions were performed using SYBR Green Real-Time PCR Master Mix (TaKaRa) on a CF96 Real-Time PCR Detection System (Bio-Rad, Hercules, California, USA). The *ACTB*, *TBP* and *TOP2B* genes were simultaneously used as internal controls for mRNA normalization. 5S rRNA served as an endogenous control for miRNA expression and was used to normalize the corresponding data. All the reactions were performed in triplicate. The 2^−ΔΔCt^ method was used to determine the relative abundance of mRNA and miRNA.

### Functional enrichment analysis for genes with DMRs

Genes with DMRs in the promoters and gene bodies were converted to human orthologous genes and submitted to the DAVID (Database for Annotation, Visualization and Integrated Discovery) web server (http://david.abcc.ncifcrf.gov/) for functional enrichment analysis of Gene Ontology (GO) and pathways. All the lists were submitted to DAVID and analyzed for significant overrepresentation of GO biological processes (GO-BP), molecular function (GO-MF) terminologies, and KEGG-pathway categories (Benjamini-corrected *P* values < 0.05 were deemed significantly different).

### Transcription analysis of mRNA and miRNA

Genome-wide gene expression analysis of six samples that corresponded to the MeDIP-seq samples was performed using an Agilent Pig Gene Expression Oligo Microarray (Version 2). The workflow of the microarray analysis was as performed previously reported^14^. Identification of differentially expressed genes and hierarchical cluster analysis were performed with the MultiExperiment Viewer. The total RNA extracted from the pigs’ LDM and PMM was pooled in equal amounts for each muscle. Approximately 45 μg of total RNA from the two tissues were used for miRNA transcriptome library preparation and sequencing. In brief, 10- to 40-nt short RNAs isolated from polyacrylamide gel electrophoresis (PAGE) were combined with adaptors (Illumina, San Diego, CA, USA) and converted to cDNA by RT-PCR. Then, the cDNA was single-end sequenced in 36 bp on a Genome Analyzer GA-2 (Illumina), per the recommended protocol for small RNA sequencing. Transcriptome data (mRNA and miRNA) have been deposited in the NCBI Gene Expression Omnibus under GEO accession numbers GSE81757 and GSE81755.

### Bisulfite sequencing PCR

The primers for bisulfite sequencing PCR (BSP) were designed by EpiDesigner software (Sequenom, USA) (primers shown in Supplementary Table S1). We treated the inspected DNA (bisulfite conversion) using the EZ DNA Methylation-Gold Kit (ZYMO Research, Irvine, CA, USA), per the manufacturer’s protocols. PCR was carried out using ZymoTaq PreMix (ZYMO Research) according to the manufacturer’s specifications, and the PCR product was purified using the DNA Clean & Concentrator - 25 Kit (Zymo Research). The PCR product was then cloned into the TA vector (Invitrogen, USA). Ten effective clones were selected for each gene and subsequently sequenced using an ABI 3730 DNA Sequencer. All of the sequences were analyzed using BiQ Analyzer V2.0 software^18^.

## Results and Discussion

### Differences in phenotypic traits between LDM and PMM

**Figure 1:**
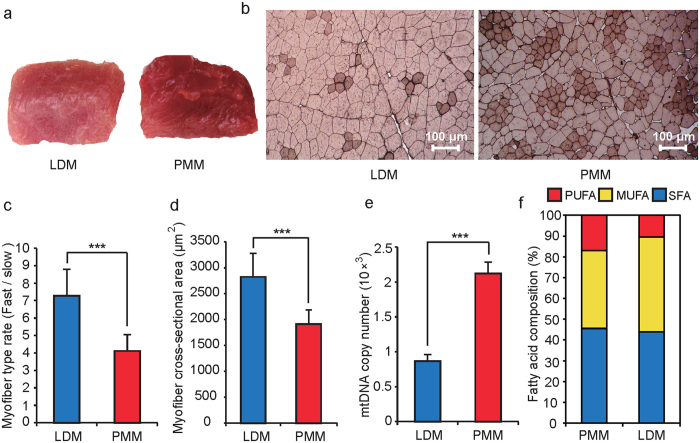
Phenotypic differences between *longissimus dorsi* muscle (LDM) and *psoas major* muscle (PMM). (**a**) The color of fresh samples of LDM and PMM. (**b**) ATPase staining of slow-twitch Type I MHCs (dark color) and fast-twitch Type II (light color) for LDM and PMM cross-sections. (**c**) Differences in the myofiber rate (Fast/slow) between LDM and PMM. (**d**) The mean myofiber cross-sectional area of LDM and PMM. (**e**) Mitochondrial DNA copy number per cell in LDM and PMM. MtDNA: Mitochondrial DNA. (**f**) The composition of fatty acids in PMM and LDM. SFA, MUMUFA and PUFA represent saturated, monounsaturated, and polyunsaturated fatty acid, respectively. Data are means ± SD. Statistical significance was calculated by Student’s t-test (n = 3 per individual). *** denotes significant differences at *P* < 0.001.

*Longissimus dorsi* muscle (LDM) and *psoas major* muscle (PMM) are typical oxidative and glycolytic muscle tissues, respectively^12^. The color of LDM had a lower redness rating than that of PMM (Fig. 1a), a result closely related to the different myoglobin contents^19^. Compared with PMM, LDM had a higher fast myofiber rate, cross-sectional area, and concentration of lactic acid and glucose. However, it had a lower mitochondrial mass (Fig. 1b–e and Supplementary Table S2), thus demonstrating why glycolytic muscle has a high glycolytic potential and fatigues more quickly than oxidative muscle^5^,^20^. Moreover, the phenotypic differentiation between the two muscle types also indicated why there are differences in meat quality characteristics, such as shear force, pH and fatty acid composition (Fig. 1f and Supplementary Tables S2 and S3). Given the consistent DNA sequence of the two different muscles in the same donor, DNA methylation and transcriptome differences are likely to underlie the different phenotypes.

### Summary of methylated DNA immunoprecipitation sequencing (MeDIP-seq) data

To explore the methylation differences between LDM and PMM, genome-wide methylated DNA immunoprecipitation sequencing (MeDIP-seq) was conducted. We obtained 897.21 million (M) reads of 43.97 gigabases (Gb) in length, approximately 15.7 times the size of the pig genome (Sscrofa 10.2), of which 42.66 Gb (89.96%) of clean reads were mapped to the pig genome. After removing ambiguously mapped reads and reads regarded as potentially duplicated clones generated via PCR amplification, there were approximately 78.49% uniquely aligned non-duplication reads (Supplementary Table S4). To acquire high-confidence methylated CpG sites for further analysis, we removed the CpG sites with read depths below ten, ultimately acquiring 34.83% of CpGs of the total genome (Supplementary Fig. S1).

**Figure 2:**
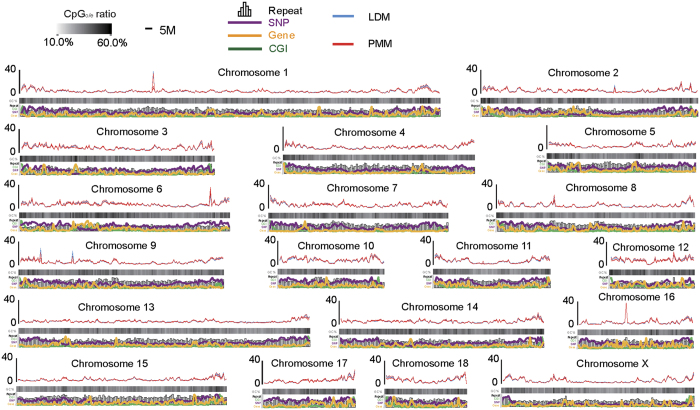
Genome-wide distribution of DNA methylation levels in pig chromosomes. The read depth was normalized to the overall average number of reads in each group. The CpGo/e ratio, SNP density, numbers of genes, repeats and CGIs were all calculated over 1-Mb sliding windows.

The distribution of MeDIP-Seq reads along chromosomes and genome features represented a genome-wide methylation pattern (Fig. 2). The methylation levels across the chromosomes were negatively correlated with chromosome length (*r* = −0.727, *P* = 4.17 × 10^−4^) and positively correlated with GC content (r = 0.804, *P* = 3.37 × 10^−5^). They also had higher than expected CpG (CpGo/e) ratios (*r* = 0.932, *P* = 1.80 × 10^−8^), repeat density (*r* = 0.638, *p* = 3.29 × 10^−3^), gene density (*r* = 0.363, *P* = 0.02), and single-nucleotide polymorphism (SNP) density (*r* = 0.804, *P* = 3.41 × 10^−5^) (Supplementary Fig. S2). Furthermore, we also observed the methylation level across the 24 defined categories of functional genomic elements. The intermediate CpG promoters (ICPs) exhibited a relatively higher methylation status than that at high CpG promoters (HCPs) and low CpG promoters (LCPs), and the DNA methylation status at the CpG island (CGI) shores was higher than that of the CGIs (Supplementary Fig. S3), thus confirming the results of previous studies^14^,^21^. The various methylation levels of genome features indicated that distinctive genome features play different roles in regulating gene expression. For example, DNA methylation at the CpG island (CGI) shores, compared with CGIs themselves, has been demonstrated to be more strongly related to gene expression^22^.

### Differential DNA methylation in the subtelomeric regions and the telomere length of LDM and PMM

**Figure 3:**
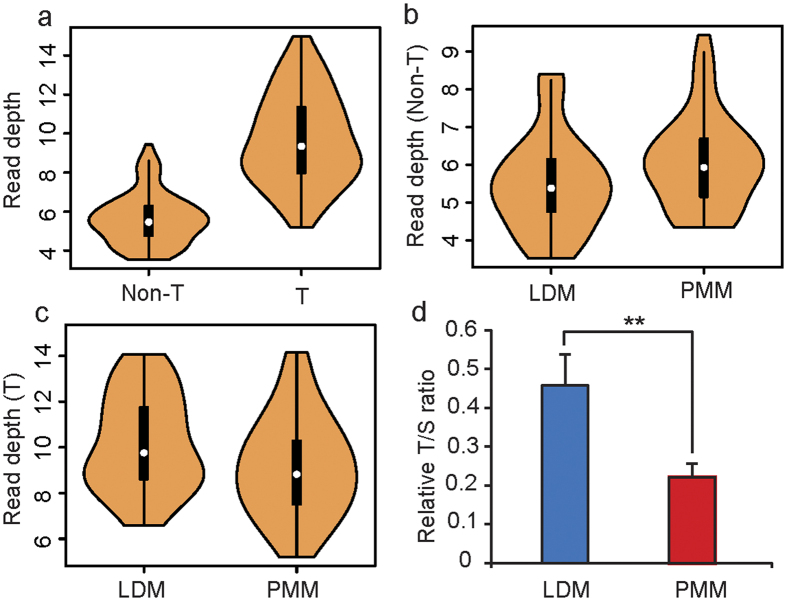
Comparison of the methylation levels between LDM and PMM across chromosomes. (**a**) Methylation levels in subtelomeric (T) and non-subtelomeric regions (Non-T) in each chromosome. Methylation levels in non-subtelomeric regions of LDM versus PMM (**b**) and methylation levels in subtelomeric regions of LDM versus PMM (**c**) in pig chromosomes. (**d**) The T/S ratio (telomere signal versus the signal of the single-copy gene) reflects the relative telomere length in LDM and PMM.

We investigated the methylation pattern of the chromosomes and found that the subtelomeric regions showed significant hypermethylation relative to non-subtelomeric regions (Fig. 3a), a result consistent with those from previous reports that the subtelomeric region of mice and pigs is heavily methylated^23^,^24^. In comparison, adjacent subtelomeric regions have a high density of CpG content^25^. Furthermore, the methylation levels of subtelomeric regions (7 Mb from each telomere) and non-subtelomeric regions exhibited distinct patterns between LDM and PMM. In non-subtelomeric regions, LDM had higher methylation levels than PMM in most (~90%, 17 out of 19) chromosomes; however, subtelomeric regions of LDM had a lower methylation status than PMM in most (~80%, 15 out of 19) chromosomes (Fig. 3b,c). Epigenetic modifications of the subtelomeric region were correlated with telomere elongation. Yehezkel *et al*. (2008) have reported that the hypomethylation of subtelomeric regions is associated with abnormally short telomeres^26^. Increasing the DNA methylation levels at the proximal subtelomere increases telomerase activity, which extends the telomere length^27^. Therefore, we measured the telomere length of PMM and LDM using qPCR and found that PMM had a relatively shorter telomere length than that of LDM (Fig. 3d); this result was consistent with the finding that PMM had a relatively lower methylation status in subtelomere regions (Fig. 3c).

Telomere length is a good candidate marker for biological age and the replication history of cells. The different telomere lengths of PMM and LDM obtained from some donors correlated with their replication capacity. The results confirmed those of previous studies, which have reported that telomere length has tissue-independent characteristics^28^. Interestingly, Sahin *et al*. have suggested that there is a direct link between telomeres and mitochondrial biology^29^. The mitochondrial production of reactive oxygen species (ROS) is a major determinant of telomere-dependent senescence; therefore, reducing mitochondrial superoxide generation reduces telomere shortening and cell replication senescence^30^. This mechanism is consistent with the finding that PMM had a higher mitochondrial content but a lower telomere length than LDM (Figs 1e and 3d). All the results indicated that the degree of replication senescence in different tissues may be correlated with methylation levels and mitochondrial metabolic activity.

### Functional enrichment analysis for genes with differentially methylated regions (DMRs)

**Figure 4:**
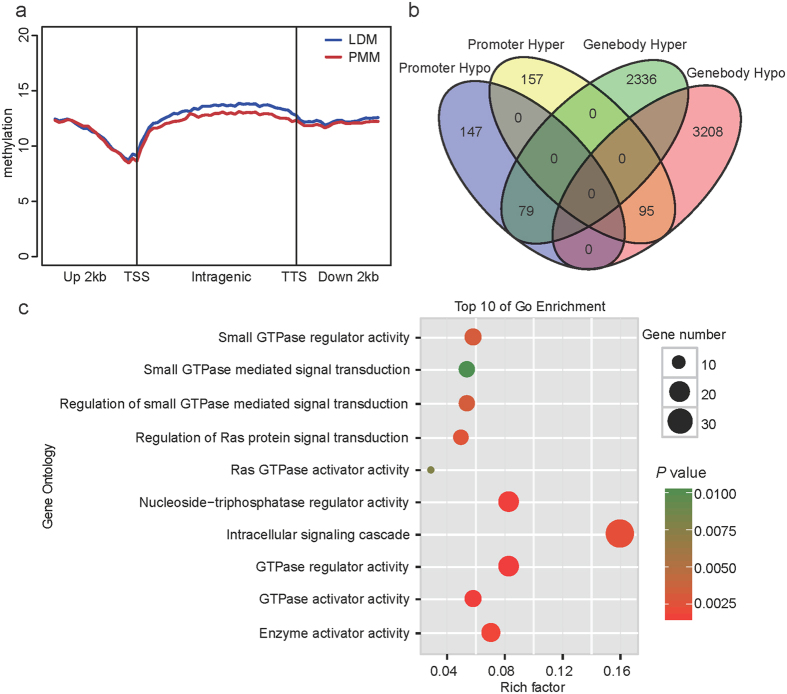
Genome-wide distribution of DMRs and function enrichment analysis. **(a)** Distribution of MeDIP-Seq reads in the gene region. The x axis indicates the position around the gene element, and the y axis indicates the normalized read number. **(b)** Venn diagram of the numbers of DMRs located in the promoters and gene bodies. **(c)** Gene Ontology (GO) categories enriched for DMRs located in the promoter regions; the top 10 GO terms are listed. The *P* values were calculated using Benjamini-corrected modified Fisher’s exact test.

The present study identified 15,633 DMRs between LDM and PMM, which represented approximately 0.91% of the length of the genome and approximately 4.01% of the total number of CpGs in the genome (Supplementary Table S5). To establish the correlation between biological replicates and experimental reliability, we performed a hierarchical clustering analysis using DMRs of each genomic element (Supplementary Fig. S4). The results indicated that these six samples clustered into two indie groups and indicated the relative epigenetic concordance within each group. To better reveal the distribution biases of DMRs in gene regions, we calculated the average methylation level in a 2-kb region upstream of the transcription start sites (TSS) and gene body and a 2-kb region downstream of the transcription termination sites (TTS) (Fig. 4a). The results indicated that differentially methylated regions were present mainly in the gene body, in agreement with the distribution of the DMRs near the gene regions; for example, there were 5,718 DMRs located in the gene body region but only 478 DMRs located in the gene promoter regions (Fig. 4b). This result indicated that the different methylation levels of gene body regions may play an important role in regulating gene expression relevant to the different biological processes between oxidative and glycolytic skeletal muscles.

DNA methylation is generally associated with the regulation of gene expression, particularly when it occurs at promoter regions^31^. To investigate the potential function of genes with differential methylation statuses in promoter regions, we performed an enrichment analysis using DAVID software. The 10 most significantly over-represented gene ontology (GO) categories were related to GTPase regulator activity and the intracellular signaling cascade (Fig. 4c). GTPases, including heterotrimeric and small GTPases, act as the essential regulatory switch in the modulation of intracellular signaling^32^. The small GTPase superfamily is classified into six subfamilies (Ras, Rac, Rho, Rab, Arf, and Ran) on the basis of their distinct functions, which regulate key cellular processes such as signal transduction, cell proliferation and cell motility^33^,^34^. These proteins function in a highly coordinated manner through signaling cascades and feedback loops involving kinases and phosphatases^35^. In the present study, we identified many genes with differential methylation statuses in promoter regions between oxidative and glycolytic skeletal muscles. These genes were enriched in the intracellular signaling cascade pathway mediated by the Ras, Rab and Rho subfamilies of small GTPase (Supplementary Table S6). Previous studies have reported that the Rab subfamily of the small GTPase activating protein TBC1D1 plays a role in the regulation of insulin tolerance, glucose utilization and glucose transport in skeletal muscle^36^. Some other small GTPase subfamilies (Rac, Rho) have also been reported to be related to the skeletal muscle signaling pathway, development and metabolism^37^,^38^. These results indicate that the differentially methylated genes related to the GTPase regulator activity pathway were the major factor that induced the different phenotypic traits between oxidative and glycolytic skeletal muscles.

Although transcriptional silencing in mammals is often associated with promoter methylation, many previous studies have demonstrated that DNA methylation in intragenic regions can alter chromatin structure and Pol II elongation efficiency in mammals and thereby influence gene expression^39^. Therefore, we performed a function enrichment analysis of genes with DMRs located in their intragenic regions. The results indicated that gene-body differently methylated genes were significantly enriched for the processes ‘ATP binding’ (368 genes, *P* = 1.83E-18), ‘protein kinase activity’ (166 genes, *P* = 9.83E-12), ‘phosphorylation’ (199 genes, *P* = 4.68E-10) and ‘phosphatidylinositol signaling system’ (30 genes, *P* = 5.12E-6). (Supplementary Fig. S5 and Supplementary Table S7). In the signaling pathways, one of the major regulatory components is the phosphorylation-dephosphorylation cascade mediated by kinases and phosphatases^40^, which, along with the phosphatidylinositol signaling system and GTPase activity pathway, contribute to the specificity of cellular responses to receptor stimulation by dynamic extracellular signaling^41^. These results indicated that the different intracellular processes between oxidative and glycolytic muscles were tightly regulated by extracellular signaling receptors, thereby leading to distinct downstream signaling networks. For example, oxidative muscles have a higher capillary density than glycolytic muscles, thus resulting in a greater oxygen and blood glucose supply than that in glycolytic muscle^42^. There are also different motor units in the muscles, and these units relate to different characteristics of neuronal cells, such as cell size, axonal diameter and conduction velocity^43^,^44^.

### Gene expression profiling and integration of DNA methylation data with expression data

**Figure 5:**
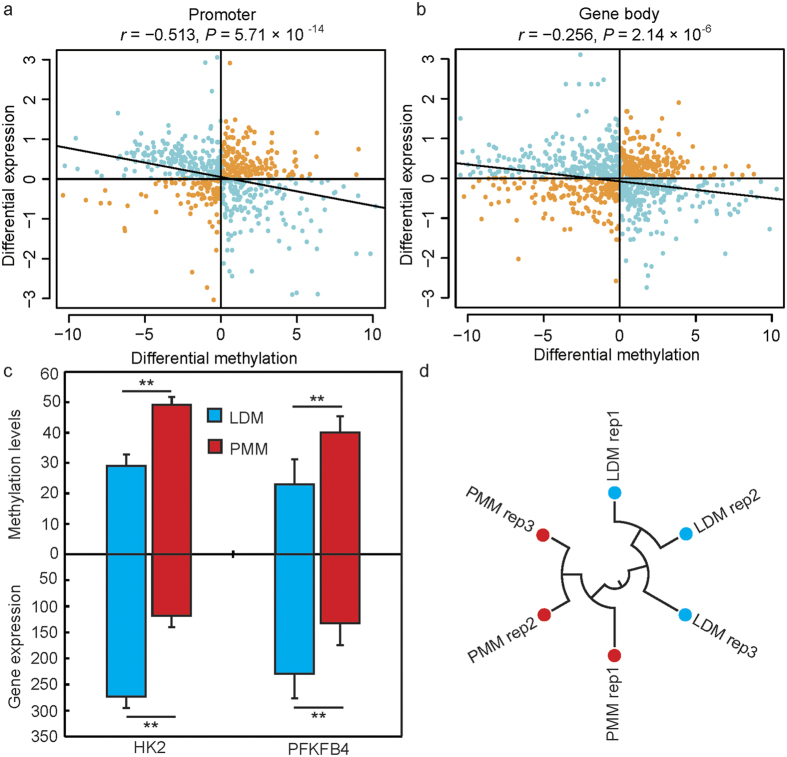
Correlation of gene expression levels with methylation profiles. Pearson’s correlation of the methylation levels of the promoter (**a**) and gene body regions (**b**) and mRNA expression. (**c**) The different methylation status and mRNA expression levels of genes related to glycolysis. *HK2*: Hexokinases 2; *PFKFB4*: 6-phosphofructo-2-kinase/fructose-2, 6-bisphosphatase 4; ** statistical significance at *P* < 0.01. (**d**) Hierarchical clustering of samples by using differentially expression genes for biological reproducibility analysis.

To obtain high-confidence gene expression data from the microarray experiments, we mapped 43,603 probes (60 mer in length) to the pig reference genome, which produced 4,983 probes uniquely mapped to exons of Ensembl genes. We then integrated the results from the expression data with the DNA methylation data. According to the correlation analysis of DMR-mRNA pairs (Fig. 5), there were negative correlations between methylation levels in the promoter and gene body regions and gene expression (*r* = − 0.513, *P* = 5.71 × 10^−14^; *r* = − 0.256, *P* = 3.186 × 10^−6^; respectively). This finding is in agreement with results from previous reports indicating that DNA methylation impedes transcriptional elongation^31^,^39^,^45^,^46^. Nevertheless, there were only 617 genes located in DMRs that were differently expressed between LDM and PMM (Supplementary Table S8). The results showed that 340 genes displayed a negative correlation, and 277 genes showed a positive correlation with mRNA expression and methylation levels. According to GO analysis, some phosphorylation (36 genes, *P* =1.25 × 10^−3^), protein kinase activity (29 genes, *P* = 1.33 × 10^−3^) and calcium ion binding (38 genes, *P* = 2.75 × 10^−3^) processes were enriched in the hypomethylated and overexpressed (or vice versa) genes between LDM and PMM (Table S9). Interestingly, oxidative phosphorylation and protein kinase activity (such as AMP-activated protein kinase) play an important role in glucose uptake and the glycolytic metabolism of skeletal muscle^36^,^47^. The calcium ion binding pathway controls the successive rise and fall of cytosolic Ca^2+^ and regulates the contraction and relaxation of muscle cells^48^. For example, *HK-2*, a key gene involved in these processes, plays an important role in encoding the first rate-limiting enzyme catalyzing the glycogen degradation reaction through glycolysis. It has previously been reported that *HK-2* is positively correlated with glycolytic potential and is less strongly expressed in slow-oxidative-type muscle^49^, a result consistent with the present findings that the high methylation status of *HK-2* resulted in lower mRNA expression levels in PMM (Fig. 5c). The phosphatase activities of PFKFB4 play important roles in increasing glucose uptake and glycolytic flux to lactate, thereby resulting in response to hypoxic environments^50^. This result also supports the possibility that the high expression of *PFKFB4* in LDM is an adaptation to anaerobic metabolism (Fig. 5c). Therefore, these biological processes are strongly linked to the differences in energy metabolism and contraction type between oxidative and glycolytic skeletal muscles.

We then performed q-PCR to validate the high throughput sequencing results. The expression patterns of six randomly selected genes were measured by q-PCR and compared with data from the microarray experiments. The results showed that the gene expression levels were highly consistent between the two methods (Supplementary Fig. S6). The reproducibility and reliability of the microarray libraries were analyzed by differential gene expression using hierarchical clustering. As shown in Fig. 5d, the three biological replicates of each sample were highly correlated with each other, and all the three libraries of each sample could be clearly assigned to one group. These results confirm the high reproducibility and reliability of the gene expression profiling in the present study.

### microRNA expression profiling and the relationship of microRNA expression to mRNA transcription and DNA methylation

**Figure 6:**
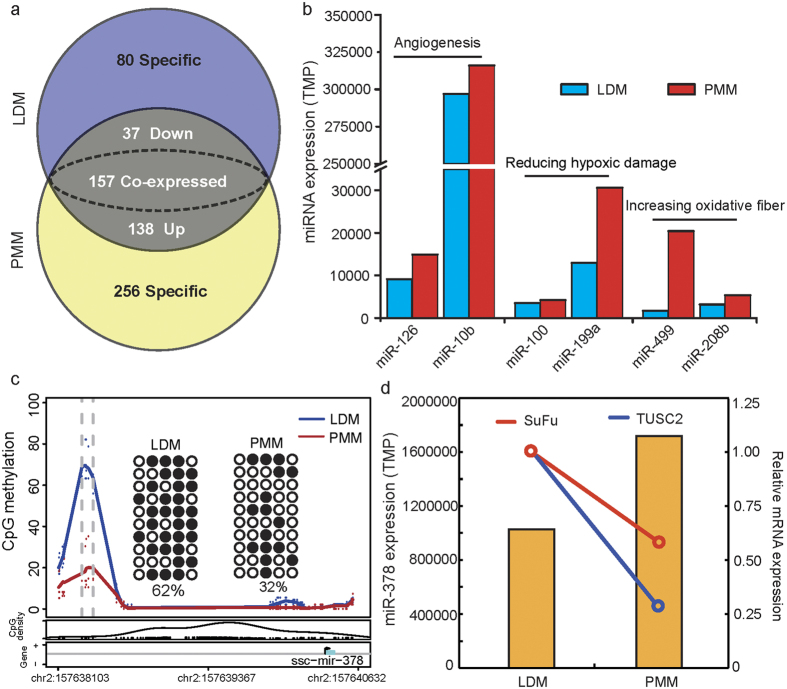
Integration analysis of microRNA expression and methylation profile. (**a**) Venn diagram of the miRNA expression profile in LDM and PMM; (**b**) Differentially expressed miRNAs involved in angiogenesis, reduced hypoxic damage and increased oxidative fiber composition; (**c**) Methylation levels of the miR-378 promoter detected by MeDIP-seq and BSP; (**d**) Expression levels of miR-378 and its target genes in LDM and PMM; *SuFu*: suppressor of fused; *TUSC2*: tumor suppressor candidate 2.

microRNA (miRNA) is an important epigenetic factor that regulates gene expression at the post-transcriptional level. We measured the global miRNA expression profiles in the same pooled RNA samples from LDM and PMM, by using transcriptome-seq. A total of 175 miRNAs (out of 668 unique miRNAs) were differentially expressed between PMM and LDM (Fig. 6a and Supplementary Table S10). Interestingly, it has previously been reported that miR-126 and miR-10b promote blood vessel formation by repressing the expression of *Spred-1* and *HoxD10*, respectively^51^,^52^. The high expression of miR-126 and miR-10b in PMM was in accordance with the higher capillary content of PMM with respect to LDM. Additionally, we have also found that the miRNAs involved in reducing hypoxic damage (miR-100 and miR-199a) and promoting slow muscle formation (miR-499 and miR-208b) are highly expressed in PMM^53^,^54^. These results partly revealed the epigenetic mechanism of the different metabolic activities and histological characteristics of the two muscle tissues.

The integration of miRNA expression data with mRNA expression profiling is useful in identifying and evaluating the effects of miRNA in regulating gene expression by inducing target mRNA degradation or translational inhibition. We therefore investigated the relationship between differences in miRNA expression between LDM and PMM and the mRNA expression levels of their potential target genes. We identified 408 and 1,232 down-regulated target genes for the up-regulated miRNAs in LDM and PMM, respectively. All the genes were significantly enriched in 373 GO terms from the biological processes analysis (Supplementary Table S11; *P* < 0.01), among which the main significantly enriched terms were intracellular signaling cascade (122 genes, *P* = 2.99 × 10^−4^), blood vessel development (17 genes, *P* = 2.33 × 10^−4^) and response to oxidative stress (29 genes, *P* = 1.28 × 10^−5^) in the reverse correlative miRNA-mRNA pairs.

Furthermore, DNA methylation in the promoters of miRNAs can also regulate miRNA transcriptional levels and lead to changes in the expression of target genes^55^. We took the site 2 kb upstream of the promoter region of miRNA and performed a correlation analysis of the depth of methylated reads and the miRNA expression levels. There was a negative correlation between methylation levels in the promoters of miRNAs and the miRNA expression levels (*r* = −2.19, *P* = 5.32 × 10^−17^; Supplementary Fig. S7), in agreement with previous studies on bovine muscle tissue^56^. However, between LDM and PMM, there were only five miRNAs that exhibited different methylation levels in their promoter regions, including miR-378, miR-181c, miR-181d, miR-139 and miR-216 (Supplementary Table S5). Interestingly, miR-378 was the second highest expressed miRNA both in LDM and PMM, but had lower methylation levels and higher expression levels in PMM. This result was confirmed in a BSP (Fig. 6b). Previous studies have reported that miR-378 regulates mitochondrial metabolism, systemic energy homeostasis and myoblast differentiation^57^,^58^. Lee *et al*. (2007) have found that miR-378 promotes angiogenesis by targeting SuFu and Fus-1 expression^59^; these findings are consistent with the present results showing lower expression of SuFu and Fus-1 in PMM (Fig. 6d). Highly methylated miR-378 in PMM, compared with LDM, may promote the growth of capillaries and mitochondrial oxidative metabolism. Additionally, the expression of miR-378, mediated by its promoter methylation status, has also been verified in cell levels^60^. Thus, the chain of methylation-miRNA-mRNA is an important molecular mechanism regulating biological function in muscles.

## Conclusions

In summary, the present study provides a comprehensive analysis of genome-wide DNA methylation and its relationship with mRNA and the miRNA transcriptomes in oxidative and glycolytic skeletal muscles. We identified differences in the DNA methylation between the two types of muscle, particularly in gene body regions. Furthermore, numerous differentially methylated genes that exhibit close relationships with the distinct phenotypic traits of oxidative and glycolytic muscle were identified. These genes mainly function in ATP binding, calcium ion binding, glycolysis metabolism and angiogenesis, thus revealing the different molecular mechanisms of metabolic activities and histological characteristics between oxidative and glycolytic muscle. This integrated analysis should serve as a valuable resource for further muscle development studies.

## Additional Information

**Accession codes:** All the high-throughput sequencing data have been deposited in NCBI’s Gene Expression Omnibus under GEO Series accession numbers GSE81766.

**How to cite this article**: Shen, L. *et al*. Genome-wide landscape of DNA methylomes and their relationship with mRNA and miRNA transcriptomes in oxidative and glycolytic skeletal muscles. *Sci. Rep.* **6**, 32186; doi: 10.1038/srep32186 (2016).

## Supplementary Material

Supplementary Table S5-S11

Supplementary Information
