# Supplementary material for: Genome-wide landscape of DNA methylomes and their relationship with mRNA and miRNA transcriptomes in oxidative and glycolytic skeletal muscles
Source: Sci Rep. 2016 Aug 26;6:32186. doi: 10.1038/srep32186 (PMC4999948; doi:10.1038/srep32186)
Supplement: Supplementary Information [file srep32186-s2.pdf]

Supplementary Information

Genome-wide landscape of DNA methylomes and their relationship with mRNA and miRNA transcriptome profiling between oxidative and glycolytic skeletal muscles

Linyuan Shen, Jinjin Du, Yudong Xia, Zhendong Tan, Yuhua Fu, Qiong Yang, Xuewei Li, Guoqing Tang, Yanzhi Jiang, Jinyong Wang, Mingzhou Li, Shunhua Zhang, & Li Zhu

On-line Supplementary Data

Supplementary Figures:

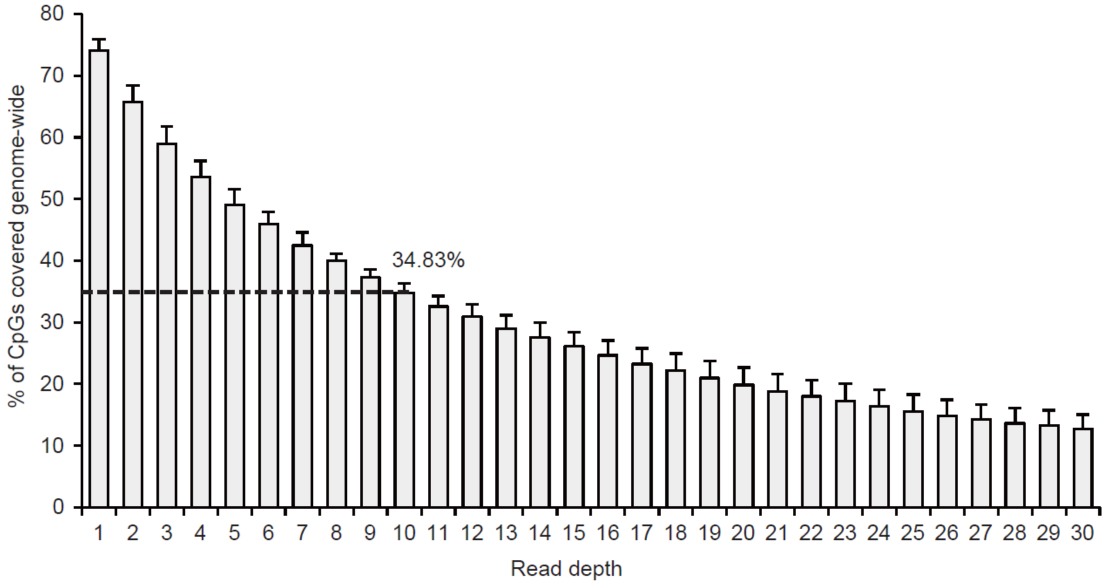

Supplementary Figure S1. Percentage of CpGs sites showing an average coverage that meets the read depth threshold over all samples. Values are the means ± s.d. (n = 6).

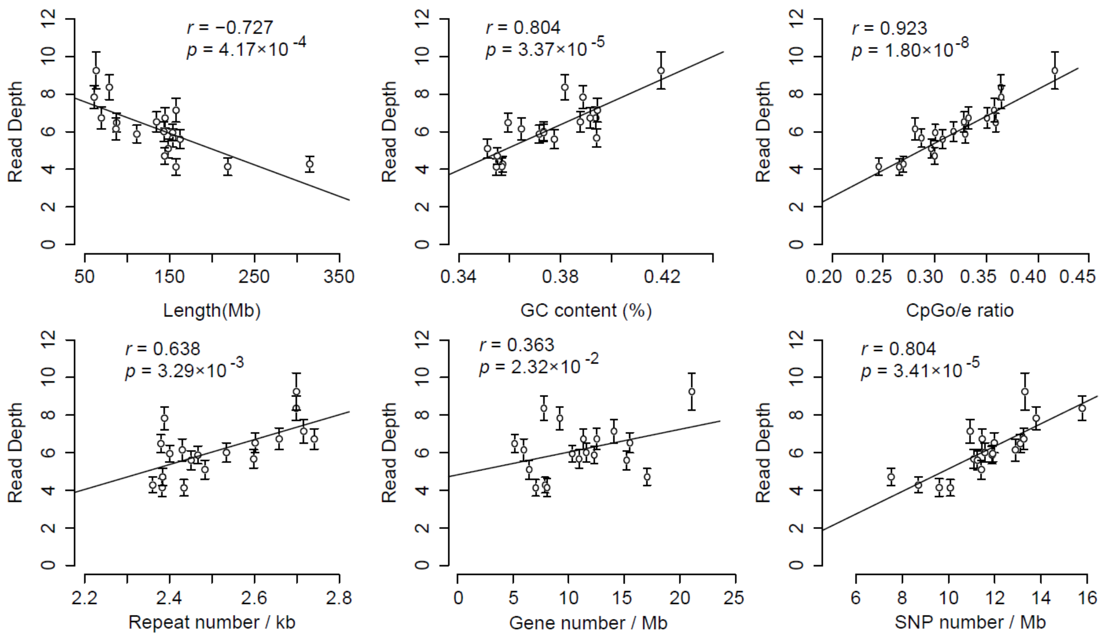

Supplementary Figure S2. Pearson's correlation between DNA methylation level and chromosomal features.

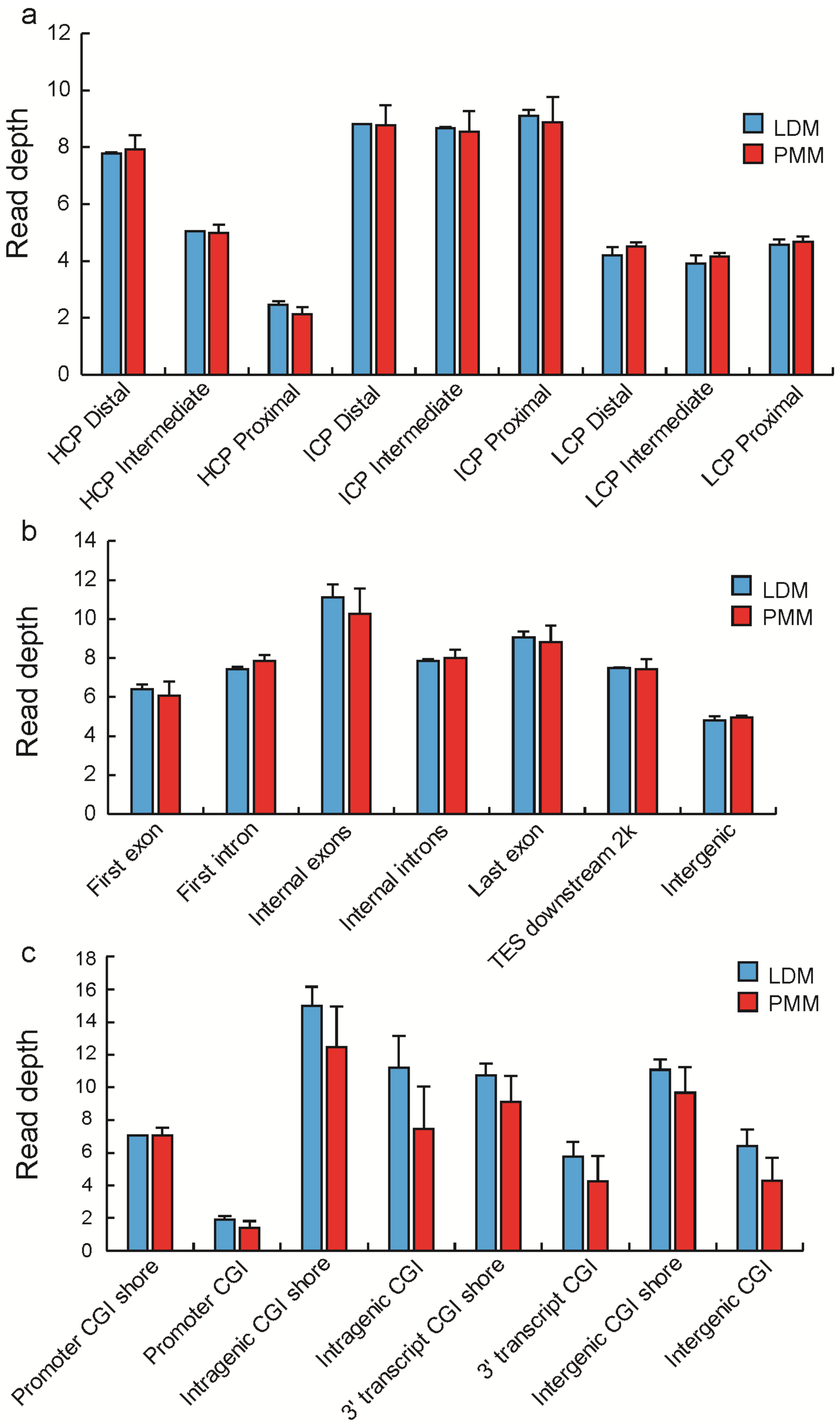

Supplementary Figure S3. Comparison of methylation levels in various of genomic elements

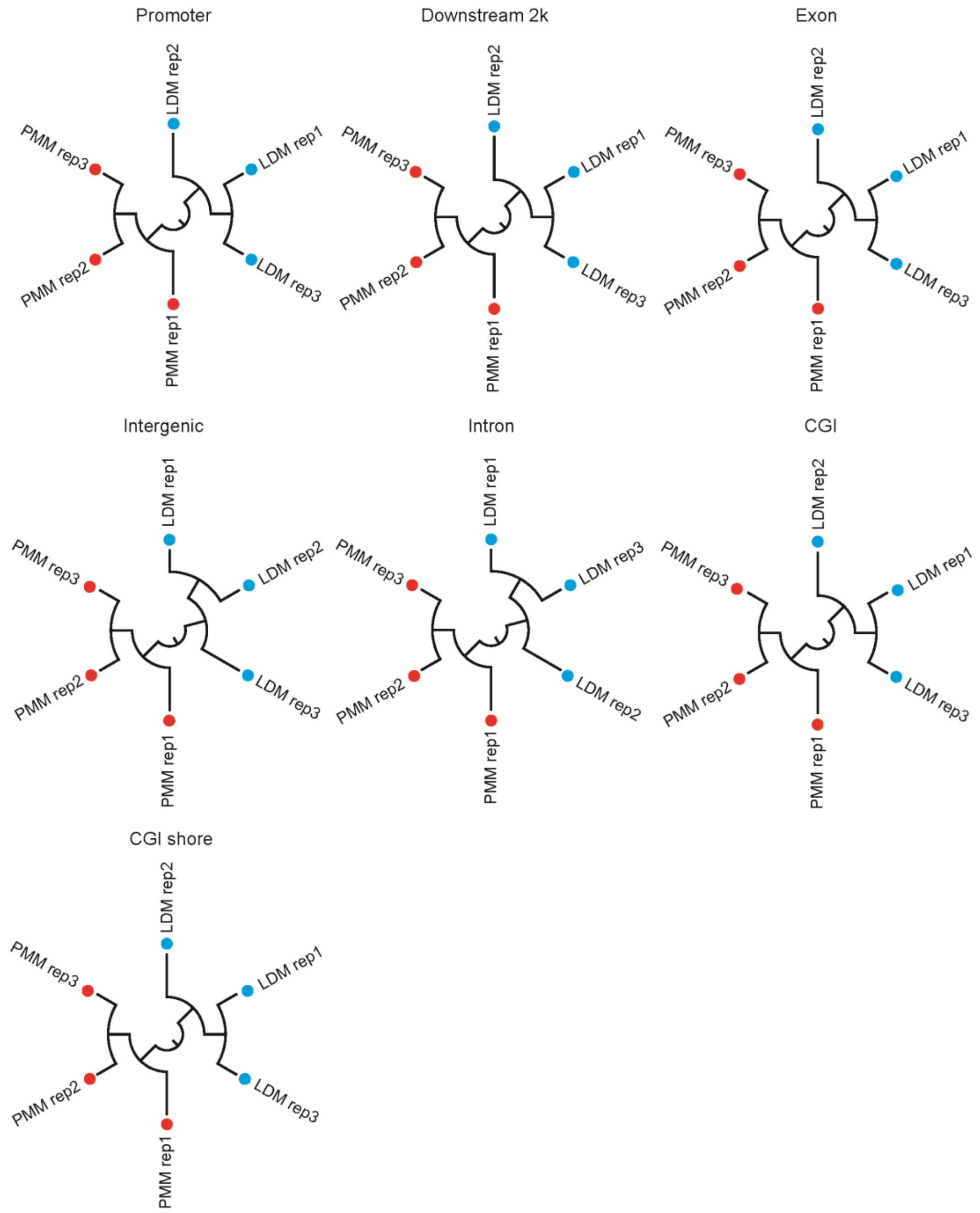

Supplementary Figure S4. Hierarchical clustering of samples using DMRs in various genomic elements. The distance metric applied for clustering was Pearson correlation across samples.

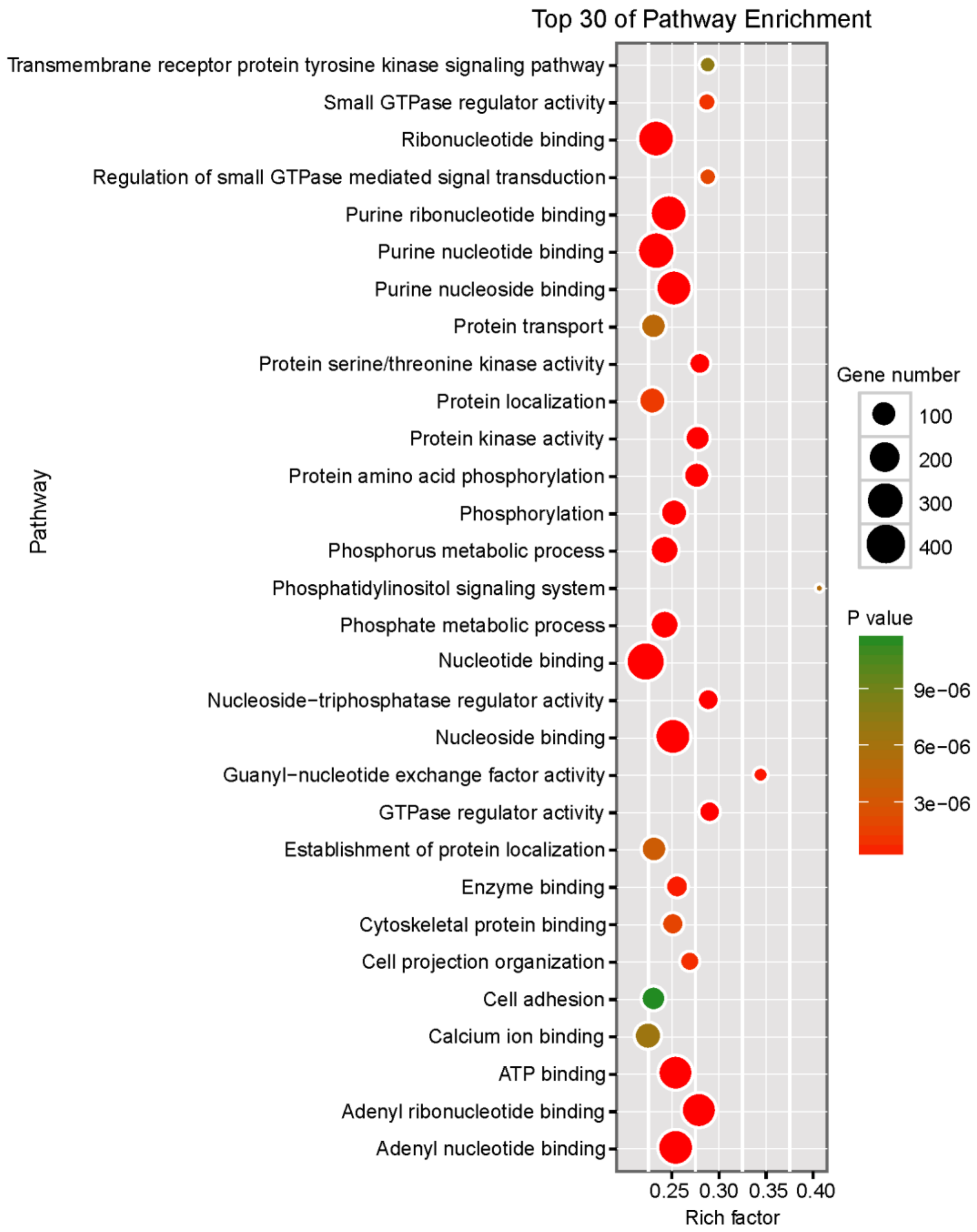

Supplementary Figure S5. Function enrichment nanlysis of DMRs in gene body regions.

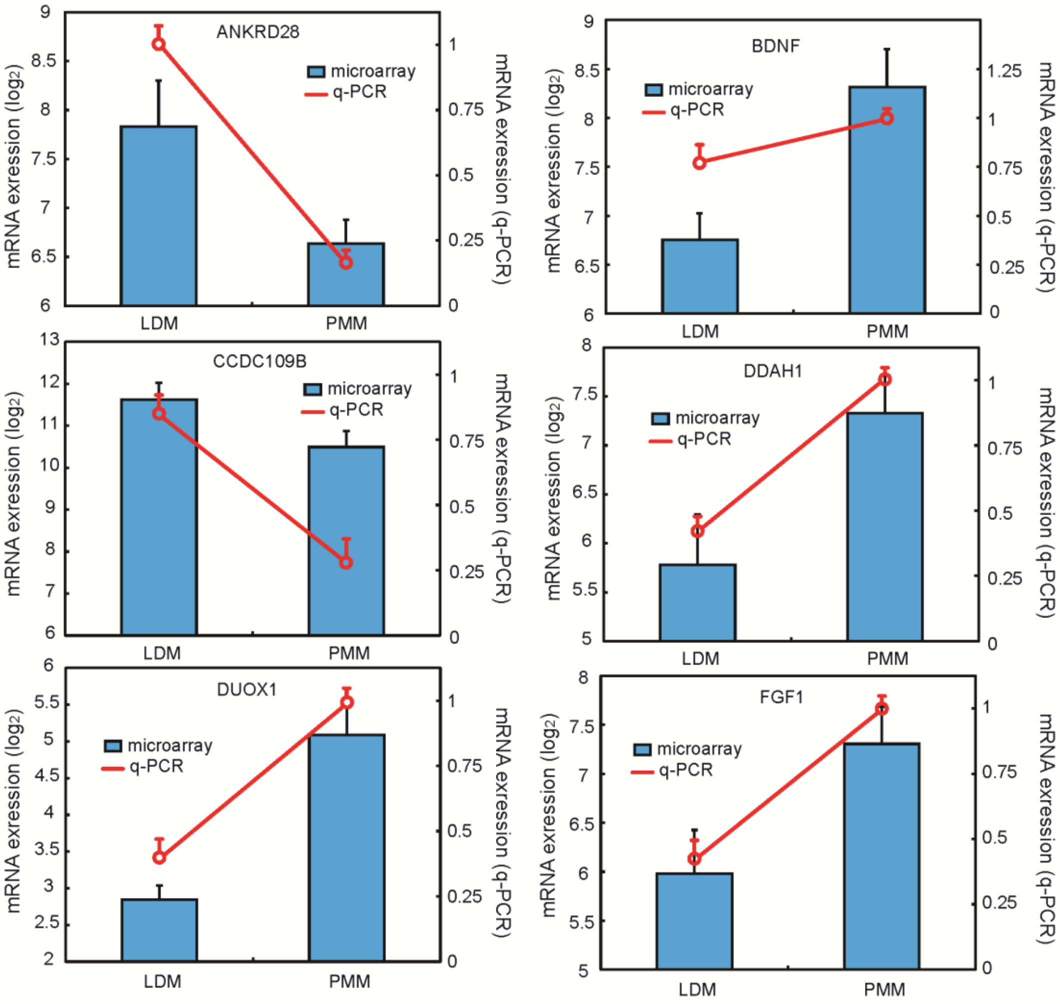

Supplementary Figure S6. Verification of mRNA expression data by microarray using q-PCR. The whole name of genes were shown in Table S1

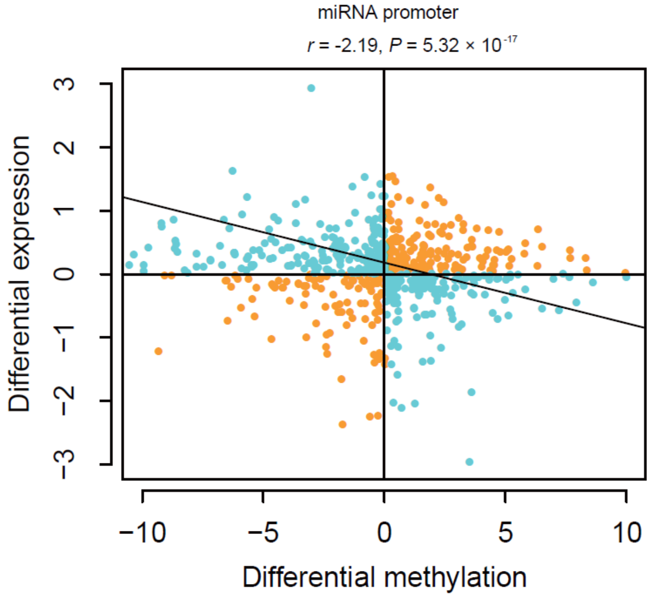

Supplementary Figure S7. The Pearson's correlation of miRNA promoter methylation levels and miRNA expression.

Supplementary Table S1. Information on primers used to perform Q-PCR and BSP.

Gene symbol	Forword primer	Reverse primer
COX1	ACTACTGACAGACCGCAACC	TCCAATGGACATTATGGCTC
GCG	GAATCAACACCATCGGTCAAAT	CTCCACCCATAGAATGCCCAGT
SuFu	ATGAGGACAGCCGGAGCAT	TTGGCGGAAGGACAGGTTT
TUSC2	AGACAATCATCACCAAGAACGG	CCTCATAGAGGATCACAGGGAAA
ANKRD28	TTACCAGTGCCTGTTTGCT	TTGTATCCTTGCTTGTCCC
BDNF	TCTTTCTGCTGGAGGAATACA	ACCCACTCGCTAATGCTGT
CCDC109B	GCCTGTCTGGGCAGTTCTA	TTCTGGGATGCTCTTGGTC
DDAH1	TTTTAAGGACTATGCGGTGTC	ATCCAATGGCAATGAGGTT
DUOX1	ACCATTGGGACTCTGTGCTG	CATGATACTCTGGCGGTTCTG
FGF1	ACGGCTCACAGACACCCAG	GCGTTTGCAGCTTCCATTC
miR-378	ACUGGACUUGGAGUCAGAAGGC	Uni-miR qPCR Primer, included in kit
BSP-378	AAAAGGTTTGTAGGTGTAAGGG	CCATCTCTAACTCTCAAAACTCC
Telomeric region primer (T)	CGGTTTGTTTGGGTTTGGGTTTGGGTTTGGGTTTGGGTT	GGCTTGCCTTACCCTTACCCTTACCCTTACCCTTACCCT
5S rRNA	GCCCGATCTCGTCTGATCT	AGCCTACAGCACCCGGTATT
ACTB	TCTGGCACCACACCTTCT	TGATCTGGGTCATCTTCTCAC
TBP	GATGGACGTTCGGTTTAGG	AGCAGCACAGTACGAGCAA
TOP2B	AACTGGATGATGCTAATGATGCT	TGGAAAAACTCCGTATCTGTCTC

Abbreviations for primers used throughout the study: COX1, cytochrome c oxidase subunit I; GCG, glucagon; SuFu,suppressor of fused homolog; TUSC2, tumor suppressor candidate 2; ANKRD28, ankyrin repeat domain 28; BDNF, brain-derived neurotrophic factor; CCDC109B, coiled-coil domain containing 109B; DDAH1, dimethylarginine dimethylaminohydrolase 1; DUOX1, dual oxidase 1; FGF1, fibroblast growth factor 1; ACTB, actin, beta; TBP, TATA-box binding protein; TOP2B, topoisomerase (DNA) II beta. Telomeric region primers used to detect the telomeric length; BSP-378 used to amplify the promoter of miR-378 for BSP reaction; 5S rRNA was used as an endogenous control for miRNA quantitative analysis. ACTB, TBP and TOP2B were used as an endogenous control for mRNA quantitative analysis.

Supplementary Table S2. Fatty acid composition in Landrace muscle tissue (%)

Fatty acid (%)	PMM	LDM
C10:0	0.05	0.00
C12:0	0.07	0.06
C14:0	1.13	1.15
C16:0	26.94	25.68
C16:1	1.12	2.31
C17:0	0.47	0.73
C17:1	0.25	0.21
C18:0	16.59	15.99
C18:2n-6	12.89	6.55
C18:3n-3	0.49	0.28
C18:3n-6	0.05	0.00
C18:n-9	36.14	43.08
C20:0	0.33	0.35
C20:2	0.60	0.47
C20:3n-3	2.87	3.01
C20:5n-3	0.01	0.03
C22n-9	0.00	0.02
C22:6n-3	0.00	0.07
SFA	43.96	45.58
MUFA	45.61	37.51
PUFA	10.43	16.91
P:S	1.03	0.83

LDM: Longissimus dorsi muscle. PMM: Psoas major muscle. SFA, MUMUFA and PUFA mean saturated, monounsaturated, and polyunsaturated fatty acid, respectively. P:S is the ratio of all PUFA to saturated fatty acid.

Supplementary Table S3. Meat quality of Landrace muscle

	LDM		PMM	
Trait	45min	24h	45min	24h
pH	6.56±0.21	5.64±0.14	6.41±0.17	5.96±0.12
L*	41.32±2.21	47.87±2.91	35.67±3.21	39.23±3.47
a*	11.29±2.15	9.38±1.37	19.27±1.57	15.73±0.84
b*	2.89±1.29	4.52±1.49	5.15±0.90	6.89±1.13
Shear force (kg)	4.67±0.55	NA	2.87±0.52	NA
Lactic acid (μmol/g)	45.06±8.32	126.32±21.42	36.43±6.51	75.62±14.64
Total sugar (mg glucose/g)	18.42±5.21	10.63±2.43	10.08±3.54	8.06±2.15

All the meat qulity was measured at postmortem 45min and 24h (4℃). LDM:Longissimus dorsi muscle. PMM: Psoas major muscle. L*, a* and b* repreasent lightness, redness and yellowness of the meat, respectively. Total sugar include the content of glycogen, glucose, and glucose-6-phosphate. All tha data are presented as the mean ± standard deviation (SD).

Supplementary Table S4. Summary of MeDIP-seq data production.

Tissue symbol	Biological replicate	Number of raw reads	Raw reads (Gb)	Number of clean reads	Clean reads (Gb)	Number of aligned reads	% aligned reads	Number of unique reads	% of unique reads
LDM	1	164,211,168	8.05	155,994,150	7.80	140,483,204	90.06%	110,292,107	78.51%
	2	154,630,448	7.58	147,049,484	7.35	132,608,309	90.18%	105,509,824	79.57%
	3	156,205,226	7.65	146,538,884	7.33	131,241,807	89.56%	102,059,717	77.76%
PMM	1	132,771,734	6.51	127,393,854	6.37	114,908,474	90.20%	91,473,739	79.61%
	2	132,486,034	6.49	126,924,070	6.35	114,200,068	89.98%	87,086,889	76.26%
	3	156,904,678	7.69	149,390,752	7.47	134,203,318	89.83%	106,131,142	79.08%
**Total**		**897,209,288**	**43.97**	**853,291,194**	**42.66**	**767,645,180**	**89.96%**	**602,553,418**	**78.49%**

The low-quality reads were filtered from the raw reads and clean reads were used in further analysis. “% aligned” is percent of clean reads aligned on the pig reference genome (version 10.2). “% unique” is percent of reads uniquely aligned over all the aligned reads. The reads with same mapping locations in each sample were taken as potentially duplicated clones created by PCR amplification during sequencing library construction, and hence were removed in the analysis.
